# Lady House Surgeons in General Hospitals

**Published:** 1901-11-30

**Authors:** 


### LADY HOUSE SURGEONS IN GENERAL HOSPITALS.

The attempts which have been made to patch up the difference at the Macclesfield Infirmary appear to have come to nothing; the medical staff have retired and the Board is left to supply their places as best it may. It is quite clear, however, that the difficulty at Macclesfield is not merely one of male *versus* female house surgeons, but is complicated by the question whether the medical staff or the board should have the selection of the officer to be appointed, and no doubt if one got to the bottom of the matter the latter would be found to be the real turning point of the disturbance. It is time, however, that the question whether a lady house surgeon is a proper person to take entire charge of a general hospital, where of necessity every kind of case must be attended to, should be decided on its merits, and we cannot but feel that it would be a great advantage if the leaders among the medical women practising in England, were to give some pronouncement which might be a guide to their juniors as to the limits (if there be such limits) which may properly be placed on the class of work which they may with propriety undertake, and might also give some indication to others, such as infirmary boards and medical colleagues, as to what may be expected from ladies who apply for medical and surgical appointments. We believe that there are enthusiastic young women who consider that having once obtained their qualifications they are equal to all emergencies. We know, on the other hand, that there are many who hold that for a young woman to pass a catheter even upon an old and feeble man is a thing which should only be done in an emergency, and that an attempt to do such a thing upon a man in his prime is to outrage all decency. In view then of the extremely diverse views which are held on this subject in different quarters, we think that the time has arrived when some definite pronouncement on the subject should be made by lady practitioners of some age, position, and experience. It is not fair that the question should have to be decided in reference to individuals. On the one hand no one wishes to place unnecessary obstacles in the path of these young ladies; and on the other those who see the difficulties naturally hesitate to speak plainly lest they should appear to impute indelicacy. The problem is one which should be decided on its own merits, and the first step to such a decision is that women themselves (not young women, fresh from medical schools, and anxious at any price to get on, but those who have seen something of the world) should say how far women may go in the treatment of male patients. If Mrs. Garrett Anderson, for example, and a few others will say that in their opinion it is right and fitting that young ladies should perform all the duties which young men now perform in attending upon male patients at general hospitals, then there will be something to go upon. As things are at present, if a scandal should arise, and if it should be asserted, as it soon would be asserted—and described in full minutiæ, too, in sensation-seeking journals — that where female house surgeons were employed things were being done which outraged all sense of decency, one party would turn round upon the board and say, " This is the sort of work which you ask young and decently brought up women to do ! " while another party would turn round upon the young ladies themselves and cry, " *O tempora ! O mores !* Such things will women do for money ! " The whole position is unfair to all concerned.

